# AMP‐activated protein kinase: a cellular energy sensor that comes in 12 flavours

**DOI:** 10.1111/febs.13698

**Published:** 2016-03-24

**Authors:** Fiona A. Ross, Carol MacKintosh, D. Grahame Hardie

**Affiliations:** ^1^ Division of Cell Signalling and ImmunologySchool of Life SciencesUniversity of DundeeScotland, UK; ^2^ Division of Cell and Developmental BiologySchool of Life SciencesUniversity of DundeeScotland, UK

**Keywords:** 2R‐ohnologue, adenine nucleotides, AMP‐activated protein kinase, cancer, energy homeostasis, LKB1, oncogene, tumour suppressor

## Abstract

The AMP‐activated protein kinase (AMPK) is a sensor of cellular energy status that is expressed in essentially all eukaryotic cells, suggesting that it arose during early eukaryotic evolution. It occurs universally as heterotrimeric complexes containing catalytic α subunits and regulatory β and γ subunits. Although *Drosophila melanogaster* contains single genes encoding each subunit, in mammals, each subunit exists as multiple isoforms encoded by distinct genes, giving rise to up to 12 heterotrimeric combinations. The multiple isoforms of each subunit are 2R‐ohnologues generated by the two rounds of whole genome duplication that occurred at the evolutionary origin of the vertebrates. Although the differential roles of these isoform combinations remain only partly understood, there are indications that they may have different subcellular locations, different inputs and outputs, and different functions. The multiple isoforms are of particular interest with respect to the roles of AMPK in cancer because the genes encoding some isoforms, such as *PRKAA1* and *PRKAB2* (encoding α1 and β2), are quite frequently amplified in tumour cells, whereas the genes encoding others, such as *PRKAA2* (encoding α2), tend to be mutated, which, in some but not all cases, may result in a loss of function. Thus, although AMPK acts downstream of the tumour suppressor liver kinase B1, and some of its isoform combinations may act as tumour suppressors that restrain the growth and proliferation of tumour cells, other isoform combinations may paradoxically act as oncogenes, perhaps by aiding the survival of tumour cells undergoing environmental stresses such as hypoxia or nutrient deprivation.

**Abbreviations**
1R/2R: one/two rounds of whole genome duplication
ACACB: acetyl‐CoA carboxylase‐2
ADaM site: allosteric drug and metabolite binding site
AMPK: AMP‐activated protein kinase
CaMKK2: calmodulin‐dependent kinase kinase‐2
CaMKKβ: calmodulin‐dependent kinase kinase‐β
CBS repeat: cystathionine β‐synthase repeat
IGF‐1: insulin‐like growth factor‐1
LKB1: liver kinase B1
MEF: mouse embryo fibroblast
NES: nuclear export sequence
RIM: regulatory subunit interaction motif
α‐AID: α subunit auto‐inhibitory domain
α‐CTD: α subunit C‐terminal domain
α‐KD: α α subunit kinase domain
β‐CBM: β β subunit carbohydrate‐binding module
βα/β ‐CTD: β α/β subunit C‐terminal domain

## Introduction

The AMP‐activated protein kinase (AMPK) is a sensor of cellular energy status expressed in essentially all eukaryotic cells, including protists, fungi, plants and animals 1, 2, 3. AMPK orthologues appear to exist universally as heterotrimeric complexes comprised of catalytic α subunits and regulatory β and γ subunits. In invertebrate species such as *Drosophila melanogaster* the α, β and γ subunits of the AMPK orthologue are encoded by single genes 4. However, in vertebrates, all three subunits exist as multiple isoforms encoded by distinct genes 5, 6, 7. In humans, the α1 and α2 isoforms are encoded by *PRKAA1* and *PRKAA2*, the β1 and β2 isoforms by *PRKAB1* and *PRKAB2*, and the γ1, γ2 and γ3 isoforms by *PRKAG1*,* PRKAG2* and *PRKAG3*. These seven subunit isoforms could potentially give rise to as many as 12 heterotrimeric combinations. Co‐expression of different combinations in mammalian cells suggests that all 12 heterotrimers can be generated (D. G. Hardie, unpublished data), whereas at least six different combinations have been generated by expression in bacteria 8. However, there are indications that specific combinations are favoured in specific cell types: for example, although skeletal muscle appears to express all seven subunit isoforms at the mRNA level 5, 6, 7, studies using isoform‐specific immunoprecipitation suggest that AMPK activity in that tissue can be accounted for by just three combinations: α1β2γ1, α2β2γ1 and α2β2γ3 9.

As yet, the question of whether these distinct heterotrimeric combinations have different functions has not received much attention, although interesting new evidence is beginning to emerge. Here, we review these findings, which suggest that the different combinations may be present at different subcellular locations, have different regulatory properties, and have different inputs and outputs. As will be discussed, this issue may be particularly important when considering the roles of AMPK in cancer.

## Origin of multiple isoforms of AMPK: evolution of 2R‐ohnologues

It is now believed that two rounds of whole genome duplication (1R and 2R) occurred early in the evolution of the vertebrates, although, in most cases, from one to three of the four gene copies produced by this process were subsequently lost 10. Approximately 20–30% of human genes occur as paralogues produced by these events, and these are known as 2R‐ohnologues in honour of Susumu Ohno who proposed the 2R hypothesis 11. The marine chordate amphioxus (*Branchiostoma floridae*) diverged from the vertebrate lineage before these gene duplication events, and is perhaps the best living proxy for the ancestral invertebrate because its genome generally contains a single pro‐orthologue for each family of vertebrate 2R‐ohnologues 12.

**Figure 1:**
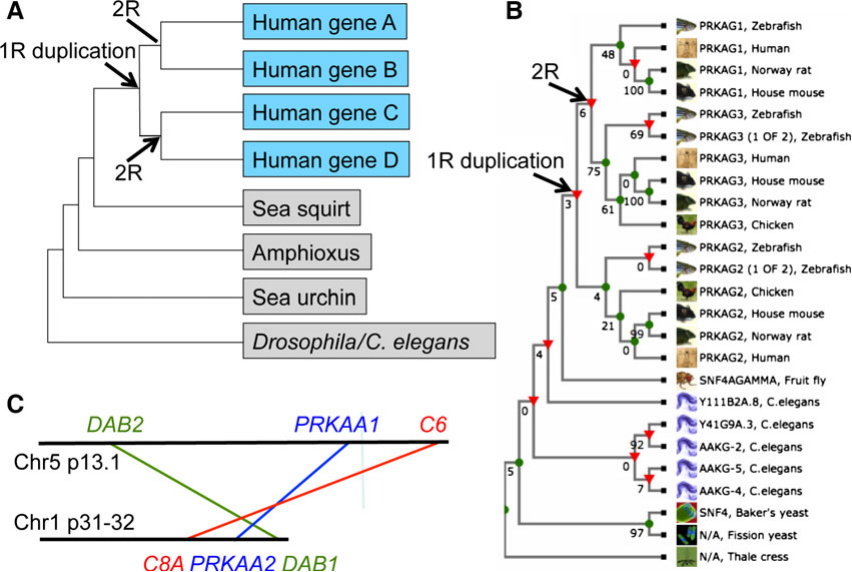
Results supporting the assignments of human AMPK subunits as members of 2R‐ohnologue families. (A) An idealized phylogenetic tree for a family of four human 2R‐ohnologues. (B) A phylogenetic cladogram derived from TreeFam (family TF313247) (http://www.treefam.org) 81 shows that the human PRKAG1, PRKAG2 and PRKAG3 proteins cluster into three paralogy groups. Although some nodes have low bootstrap values, the tree topology is consistent with a single invertebrate pro‐orthologue giving rise to two genes in the 1R during early vertebrate evolution: one of these genes then generated *PRKAG1* and *PRKAG3* in the 2R, whereas duplication of the other generated *PRKAG2* and a fourth gene that has been lost (no *PRKAG1* gene could be identified in chickens). A third genomic duplication (3R) that occurred in the last common ancestor of the teleost fish may explain the additional *PRKAG2* and *PRKAG3* genes in zebrafish. Draft sequences of pro‐orthologues of AMPK subunits in *B. floridae* (an amphioxus species) have the UniProt identifiers: alpha (C3YCL4), beta (C3Y0T7) and gamma (C3YBW1). (C) Simplified map of the regions of chromosomes 5 and 1 that share synteny and contain the *PRKAA1* and *PRKAA2* genes, respectively; *DAB2* and *DAB1* and *C6* and *C8A* are also pairs of 2R‐ohnologues.

**Figure 2:**
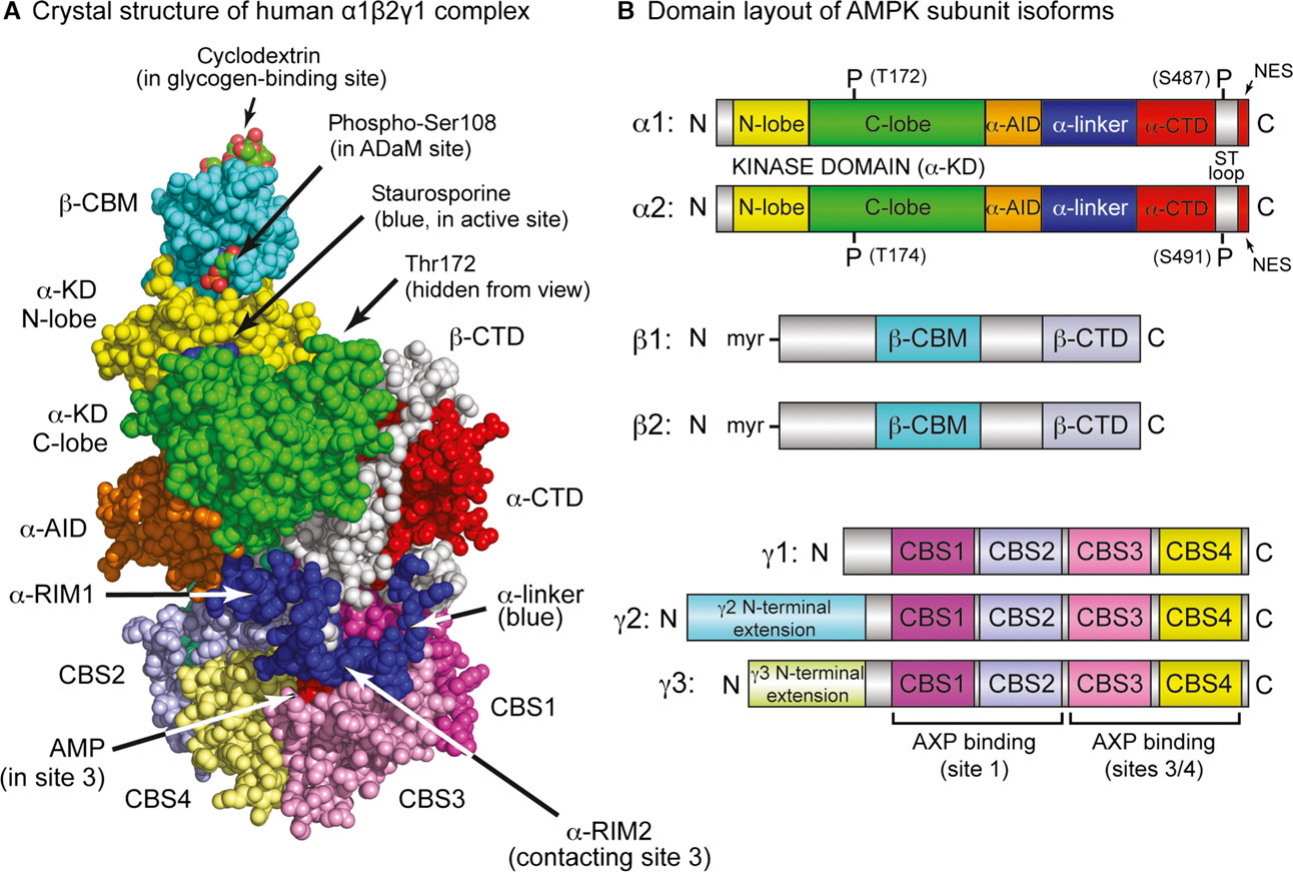
(A) Structure of the human α1β2γ1 complex and (B) domain diagrams for the human subunit isoforms. Atomic coordinates in (A) are from Protein Data Bank entry: 4RER 21 and the model was rendered in space‐filling mode in pymol, version 1.7.4.2 (Schrödinger, LLC, New York, NY, USA). The glycogen‐binding and catalytic sites in this structure are occupied by β‐cyclodextrin (C atoms, green; O, red) and staurosporine (blue); the ADaM site was empty but the position of phospho‐Ser108 (C, green; O, red) indicates its location. AMP was bound in sites 1, 3 and 4, although only that in site 3 is visible (red); the other two are around the back of the γ subunit in this view. Phospho‐Thr172 is also round the back in this view. The domain diagrams in (B) are drawn approximately to scale; domains referred to in the text are given similar colour coding in (A) and (B).

The criteria to assign paralogous vertebrate genes as 2R‐ohnologues are: (a) they are typically on different chromosomes, in blocks called paralogons that retain the gene content and order of the ancestral region from which they derive; (b) there should be a single pro‐orthologue in amphioxus in a chromosomal region showing evidence of synteny with up to four corresponding vertebrate paralogons; (c) the phylogenetic tree should have a symmetrical topology arising because the 1R generated two precursor genes, which in turn were both duplicated simultaneously in the 2R to generate four vertebrate genes relative to a single invertebrate gene (Fig. 1A); and (d) the paralogues should share similar domain architectures. Because of the 500 million years of genome rearrangements, mutations and losses since the 2R occurred, few 2R‐ohnologue families fit these criteria perfectly, although there are sufficient collective data to confidently designate the α, β and γ subunits of AMPK as three families of 2R‐ohnologues. As an example, Figs 1B, 1C and 2B illustrate criterion (c) for the γ subunits, criterion (a) for the α subunits, and criterion (d) for all three subunits, respectively. This is in keeping with the observation that the few thousand families of 2R‐ohnologues retained in modern‐day vertebrates are highly enriched in genes encoding regulatory proteins, including cytokines, growth factors, ion channels, receptors, G proteins, regulated metabolic enzymes, transcription factors and protein kinases 13, 14, 15, 16. One implication of this is that the 2R‐WGD provided an evolutionary leap in cellular communication; a quadrupling of the signalling pathways available to our last common invertebrate ancestor may have enabled the great increase in complexity and variety of the vertebrates that followed.

## Canonical regulation of AMPK and structure of heterotrimeric complexes

Mammalian AMPK complexes sense cellular energy status by monitoring the levels of AMP, ADP and ATP. Any rise in the ADP : ATP ratio, indicating a falling cellular energy (analogous to a flat battery), is converted by the adenylate kinase reaction into a much larger rise in AMP : ATP 17. The latter appears to be the primary signal that switches on AMPK, although increases in ADP may have a secondary effect 17, 18, 19, 20. Once activated, AMPK attempts to restore energy homeostasis by switching on alternate catabolic pathways that generate ATP, at the same time as switching off energy‐consuming processes, including cell growth and proliferation. A recent review 3 listed over 60 well‐validated direct targets for AMPK phosphorylation, and it is likely that the list will eventually extend into the hundreds.

Before considering the differential roles of specific isoforms, we review the features common to all heterotrimeric AMPK complexes in mammals. Figure 2A shows the structure of the human α1β2γ1 heterotrimer 21, which is similar to earlier structures for the α2β1γ1 22 and α1β1γ1 23 complexes, while Fig. 2B shows the layout of domains and other points of interest on the seven human subunit isoforms.

### Domain layout of the α subunits

The α1 and α2 subunits contain N‐terminal kinase domains (α‐KD) typical of the eukaryotic protein kinase family, with the active site (occupied in Fig. 2A by the kinase inhibitor staurosporine) in the cleft between the small N‐lobe and larger C‐lobe. Phosphorylation by upstream kinases of a conserved threonine within the activation loop (usually referred to as Thr172, although the numbering varies in different species and isoforms) can increase the kinase activity by > 100‐fold. The major upstream kinases phosphorylating Thr172 are the tumour suppressor kinase liver kinase B1 (LKB1) in complex with the accessory subunits STRAD and MO25, and the Ca^2+^/calmodulin‐dependent protein kinase CaMKK2 (CaMKKβ) 1, 2, 3. The α‐KD is followed by the auto‐inhibitory domain (α‐AID), a compact bundle of three α‐helices. In structures of α‐KD:α‐AID constructs, which invariably have a low activity 8, 21, 24, the α‐AID appears to inhibit the α‐KD by binding to its N‐ and C‐lobes on the ‘back’ side (i.e. opposite to the active site), holding it in a less active conformation. By contrast, in active conformations such as that shown in Fig. 2A, the α‐AID has rotated away from the N‐lobe and binds to the γ subunit instead. The α‐AID is followed by a flexible α‐linker that connects it to the globular C‐terminal domain (α‐CTD); this linker is crucial in regulation by adenine nucleotides, as is discussed further in the section on γ subunits below.

### Domain layout of the β subunits

A conserved MGNXXS sequence at the N‐terminus of β subunits from the animal kingdom fits the consensus for N‐myristoylation, and the human β1 and β2 subunits are indeed myristoylated on Gly2 after removal of the initiating methionine 25. When wild‐type β1, or a non‐myristoylated (G2A) mutant, was co‐expressed with GFP‐tagged α1 and γ1 in COS7 cells, glucose starvation caused the fluorescence associated with the wild‐type, but not the mutant, to shift from a diffuse cytoplasmic distribution to perinuclear speckles 25. Although these speckles were not identified, it is tempting to speculate that they represent lysosomes because glucose starvation has been reported to cause relocalization of AMPK to lysosomes as a result of its association with a complex containing AXIN and the resident lysosomal protein LAMTOR1. Because AXIN also binds LKB1, this relocalization to the lysosome is proposed to increase Thr172 phosphorylation and activation of AMPK in response to glucose starvation 26. An alternative role of N‐myristoylation of the β subunits, not necessarily mutually exclusive, was reported by Liang *et al*. 27, who proposed a role for AMPK in the selective removal of damaged mitochondria by autophagy, and suggested that this requires myristoylation of the β1 subunit to assist in binding of AMPK to mitochondrial membranes.

The myristoylated N‐termini are followed by variable sequences of approximately 100 amino acids; the structures of these regions are not known because all heterotrimer structures were determined with truncated β subunits lacking them. They are followed by the carbohydrate‐binding module (β‐CBM), which is connected by a linker that is poorly resolved in crystal structures to the C‐terminal domain (β‐CTD). The latter forms the core of the heterotrimeric complex, bridging the α and γ subunits, whereas, in all structures of active heterotrimers, the β‐CBM perches on top of the N‐lobe of the α‐KD (Fig. 2A). The β‐CBM is a member of the CBM48 family 28, whose members comprise noncatalytic domains that bind polyglucans such as starch or glycogen, usually found as components of enzymes that metabolize such polysaccharides. In mammals, the β‐CBM causes a proportion of cellular AMPK to bind to glycogen 29, 30; the function of this is not well understood, although it would co‐localize AMPK with glycogen synthase, both isoforms of which (GYS1/GYS2) are bound to glycogen particles and are inactivated by AMPK phosphorylation 31, 32.

The β‐CBM in mammalian AMPK has a second function, in that the cleft between it and the N‐lobe of the α‐KD forms the binding site for ligands such as A769662 and 991, which are synthetic compounds derived from high‐throughput screens that searched for allosteric AMPK activators 22, 23, 33. This binding cleft is stabilized by autophosphorylation of Ser108 on the β‐CBM (Fig. 2A), whose phosphorylated side chain interacts with two conserved lysines on the α‐KD N‐lobe 22. It has been termed the allosteric drug and metabolite (ADaM) binding site, although no natural metabolite derived from mammals has yet been shown to bind there. However, salicylate, the natural plant product used as a medicine ever since ancient times, and from which acetyl salicylic acid (aspirin) was derived, activates AMPK by binding to the ADaM site 23, 34.

### Domain layout of the γ subunits

The γ subunits contain variable N‐terminal domains that will be discussed further below, followed by four tandem repeats [cystathionine β‐synthase repeats 1–4 (CBS1–CBS4)] of a sequence motif known as a CBS repeat. These also occur (although usually as just two repeats) in a small number of other proteins in the human genome where they have been shown to bind regulatory ligands containing adenosine, such as ATP or *S*‐adenosyl methionine, in the cleft between each pair of repeats 35. In the AMPK‐γ subunits, the four repeats fold into a disk‐like shape with one repeat in each quadrant, generating four pseudosymmetrical clefts in the centre that comprise the binding sites for the regulatory nucleotides AMP, ADP and ATP 35. However, crystal structures suggest that only three of these, designated site 1 (between CBS1 and CBS2) and sites 3 and 4 (between CBS3 and CBS4) are ever occupied 19, 22, 36.

Binding of AMP to the γ subunit activates AMPK by three mechanisms 17: (a) allosteric activation; (b) promotion of Thr172 phosphorylation by LKB1; and (c) inhibition of Thr172 dephosphorylation by protein phosphatases. Although the roles of the three AMP‐binding sites are not completely understood, the critical site for mechanisms (a) and (c) appears to be site 3. The α‐linker mentioned earlier contains two conserved sequence motifs termed α‐RIM1 and α‐RIM2 (where RIM is the regulatory subunit interaction motif). In all three structures of active human heterotrimers 21, 22, 23, which have AMP bound at sites 1, 3 and 4, α‐RIM1 interacts with the surface of CBS2 close to the unoccupied site 2, whereas α‐RIM2 interacts with the surface of CBS3 via residues that also interact with the AMP bound in site 3 (Fig. 2A). The α‐AID and α‐linker appear to form a flexible hinge between two regions of the heterotrimer termed the catalytic module (containing the α‐KD and β‐CBM; Fig. 2A, top left) and the nucleotide‐binding module (containing the α‐CTD, β‐CTD and γ subunit; Fig. 2A, bottom right). A current model based on various structural, biophysical and mutational analyses 21, 22, 23, 24, 37, 38, 39, 40 suggests that AMP binding at site 3 promotes the interaction between the α‐RIM2 sequence and CBS3, causing the α‐linker to ‘pull’ the α‐AID away from its inhibitory interaction behind the α‐KD and thus explaining allosteric activation by AMP. Conversely, when ATP is bound at site 3, the α‐linker dissociates from the γ subunit, allowing the α‐AID to rotate back into its inhibitory position behind the α‐KD. The partial separation of the catalytic and nucleotide‐binding modules allowed by this release of the α‐linker may also make Thr172 more accessible to protein phosphatases, explaining how ATP binding relieves the protective effect of AMP on Thr172 dephosphorylation [mechanism (c)]. This model leaves open the functions of nucleotide binding at sites 1 and 4, although, as a result of the three binding sites lying very close together at the centre of the γ subunit, it is inevitable that there will be interactions between them.

## AMPK: tumour suppressor or oncogene?

We now briefly consider the role of AMPK in cancer, which will become relevant to our subsequent discussion of the functions of the different subunit isoforms. AMPK is known to inhibit cell growth, not only directly by inhibiting biosynthetic pathways such as lipid, glycogen and rRNA biosynthesis, but also indirectly by inactivating a key signalling node that promotes cell growth, the mechanistic target‐of‐rapamycin complex‐1 1, 2, 3. AMPK can also inhibit cell proliferation by causing a G1 cell cycle arrest 41, 42. Given these potential cytostatic effects, it had been widely assumed that AMPK exerts many, if not all, of the tumour suppressor functions of its upstream kinase, LKB1. Supporting this are findings showing that whole‐body knockout of AMPK‐α1 in mice accelerated the development of lymphomas driven by over‐expression of the oncogene Myc in B cells 43. If AMPK is a tumour suppressor, there would also be selection pressure for it to be down‐regulated in cancers. This does indeed occur by various mechanisms in different cancers, including phosphorylation of AMPK‐α1 at Ser487 by Akt, which inhibits its subsequent phosphorylation at Thr172 and activation by LKB1 44, and degradation of AMPK‐α1 following polyubiquitylation by the E3 ligase TRIM28, which is targeted to AMPK by MAGE‐A3/‐A6; the latter were originally defined as tumour antigens normally only expressed in testis, but also aberrantly re‐expressed in many cancers 45.

Despite this, it now appears that a complete loss of AMPK function, particularly in solid tumours, may limit their viability by reducing their tolerance to stresses such as hypoxia, glucose deprivation or oxidative stress. The first evidence for this view came from studies of H‐Ras‐transformed mouse embryo fibroblasts (MEFs) with/without a double AMPK‐α1/α2 knockout, where it was found that the knockout cells grew normally *in vitro* but failed to grow *in vivo* in immunodeficient mice 46. The inference was that the cells were viable in the rich medium of a culture dish but not in the tougher environment *in vivo,* where the supply of oxygen and nutrients may often be limiting. More recently, knocking down or knocking out AMPK has been shown to reduce the growth or viability of tumour cells in several different *in vitro* and *in vivo* models; a more comprehensive coverage is provided elsewhere 47, 48, 49, 50. These findings have resulted in a modified view of the role of AMPK in cancer. Thus, AMPK may initially limit the rapid growth and proliferation of incipient tumour cells, such that they would be under selection pressure to down‐regulate the pathway and reduce its restraining influence. However, a complete loss of AMPK might paradoxically create a severe disadvantage to the tumour cells, rendering them more vulnerable to the stresses that occur as they outgrow the capacity of their blood supply to deliver oxygen and nutrients. This new synthesis of the roles of AMPK in cancer is considered further below.

## Differential functions of complexes containing different subunit isoforms

### Differences between α1 and α2 (PRKAA1 and PRKAA2)

The sequences of the human α1 and α2 isoforms are 90% identical within the kinase domains, and early work using peptide substrates suggested that their substrate specificities were similar, if not identical 51. Most cell types express both isoforms, although cells in the haemopoietic lineage only express α1. This helps to explain the most obvious phenotype of α1 knockout mice, which concerns erythrocytes. Erythrocytes lacking α1 have rigid and nondeformable plasma membranes, and are therefore more susceptible to damage by shear stress during passage through capillaries, triggering severe anaemia 52. By contrast, α2 knockout mice have a quite different phenotype: they are insulin‐resistant and glucose‐intolerant, apparently as a result of an overactive sympathetic nervous system and consequent hyper‐secretion of adrenaline (epinephrine), which inhibits insulin secretion and action 53. Because several different hormones inhibit AMPK in the ventromedial hypothalamus and simultaneously activate the sympathetic nervous system 54, 55, 56, the phenotype of global α2 knockout might be caused by its loss in hypothalamic neurones. Although whole body α1 or α2 knockouts are therefore viable albeit with distinct phenotypes, simultaneous knockout of both α subunits causes embryonic lethality 57. Thus, although the functions of α1 and α2 are distinct, each can compensate for loss of the other, except perhaps in those few cell types where only one is expressed.

There is some limited evidence showing that the two catalytic subunit isoforms have different subcellular locations. This is a neglected topic but is important because it is likely that much of the substrate selectivity of the isoforms derives from their distinct subcellular locations, rather than their intrinsic specificity. Although neither isoform is exclusively localized to the nucleus, α2 appears to be more enriched in the nucleus than α1 in several cell types, including skeletal muscle 58, 59, 60. In the mouse C2C12 muscle cell line, the adipokines leptin and adiponectin caused activation and nuclear translocation of α2 but not α1 61. Contrasting results were obtained in mouse liver, where α1 expression within the nucleus was found to vary in a circadian manner, correlating with expression at the mRNA level of β2 but not β1, whereas the expression of α2 in the nucleus was constant 62. Exactly how AMPK complexes shuttle between the cytoplasm and the nucleus remains unclear, although a putative nuclear localization sequence in α2, which is not fully conserved in α1, has been identified 61. The α2 isoform also has a well‐defined and functional nuclear export sequence (NES) at its extreme C‐terminus 63; a putative NES is also present at the same position in α1, although it has not yet been shown to be functional.

AMPK has also been reported to localize in stress granules, regions of cytoplasm that form in cells experiencing metabolic or oxidative stress, which contain translationally arrested mRNAs and are nucleated by the protein G3BP1 (Ras GTPase activating protein‐binding protein‐1). Interestingly, α2, but not α1, has been reported to co‐localize with stress granules, and this may be the result of a direct interaction with G3BP1 64.

The two isoforms also differ in their cross‐talk with other signalling pathways. As mentioned above, AMPK‐α1 is phosphorylated at Ser487 (human numbering) by the protein kinase Akt, a key downstream mediator of the insulin/IGF‐1 signalling pathways 44, 65. Ser487 is located within the ST loop, a serine/threonine‐rich sequence of approximately 50 residues that lies just before the NES at the C‐termini of both α1 and α2 (Fig. 2B). This loop is not present in AMPK orthologues from most nonvertebrates and, in its unphosphorylated form, appears to be largely unstructured because it is not resolved in any of the heterotrimer structures. However, phosphorylation of Ser487 by Akt inhibits subsequent Thr172 phosphorylation and consequent activation by LKB1, leading to reduced AMPK activation in various tumour cell lines in which Akt has been hyper‐activated by loss of the tumour suppressor PTEN 44, 65. The residue equivalent to Ser487 on human α2 is Ser491, which (unlike Ser487) is an extremely poor substrate for Akt and is rapidly auto‐phosphorylated instead 44. Thus, although the ST loop may be a key locus for cross‐talk between AMPK and other signalling pathways, this appears to occur in an isoform‐specific manner.

**Figure 3:**
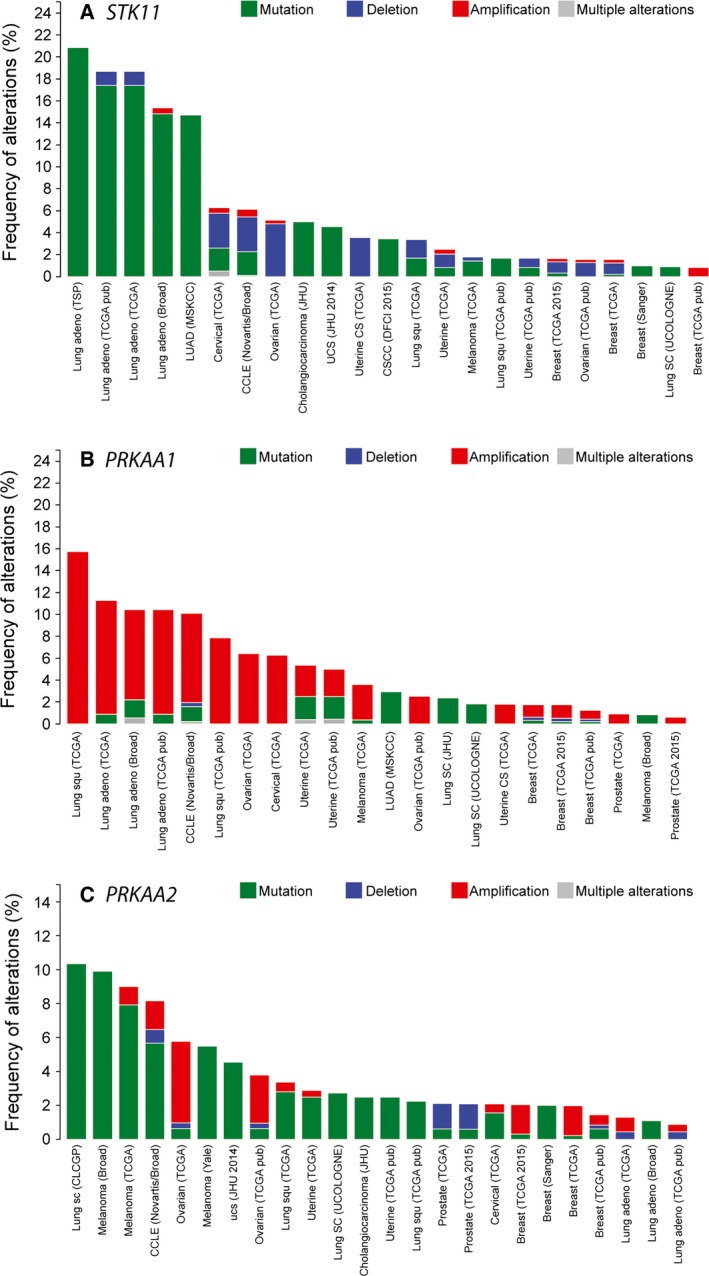
Frequency of alterations in (A) *STK11* (encoding LKB1), (B) *PRKAA1* (encoding AMPK‐α1) and (C) *PRKAA2* (encoding AMPK‐α2) displayed using cBioPortal [66,67] . Data are from a selected group of 43 cancer genome studies, and only those with alterations are displayed. Note how the alterations in *STK11* are most frequently mutations (although deletions are particularly prevalent in cervical, ovarian and uterine cancers), whereas *PRKAA1* is quite frequently amplified. With some exceptions, alterations in *PRKAA2* are mostly mutations, and the frequency is lower (note different scales on the *y*‐axes).

**Figure 4:**
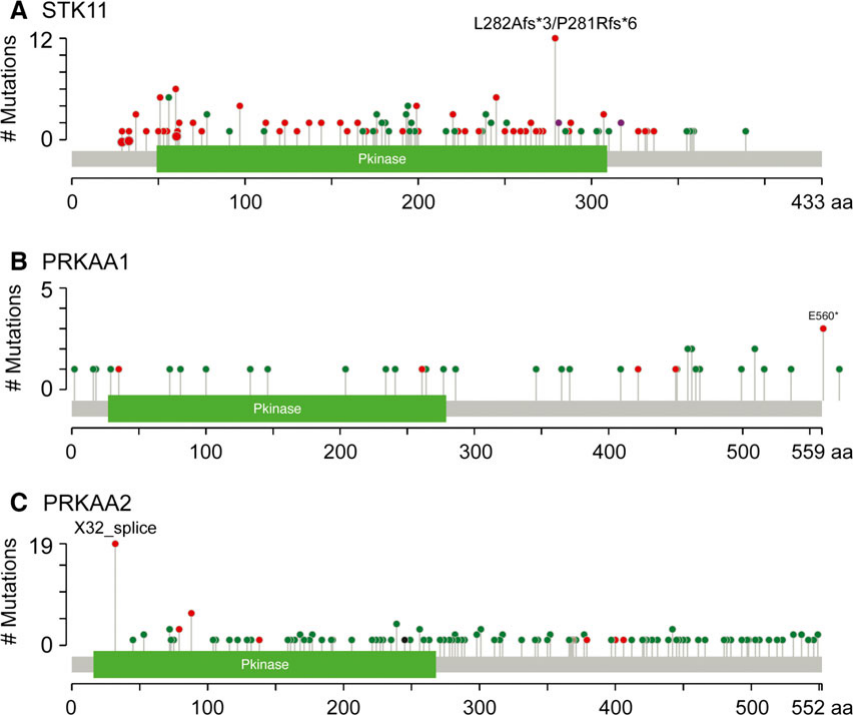
Summary of nonsynonomous mutations in (A) *STK11*, (B) *PRKAA1* and (C) *PRKAA2* in the same set of cancer studies as in Fig. 3. Red dots indicate the positions of nonsense, frameshift or splicing mutations that would give rise to truncated or aberrantly spliced proteins, whereas green dots are the positions of missense mutations, which are less likely to cause a loss of function.

**Figure 5:**
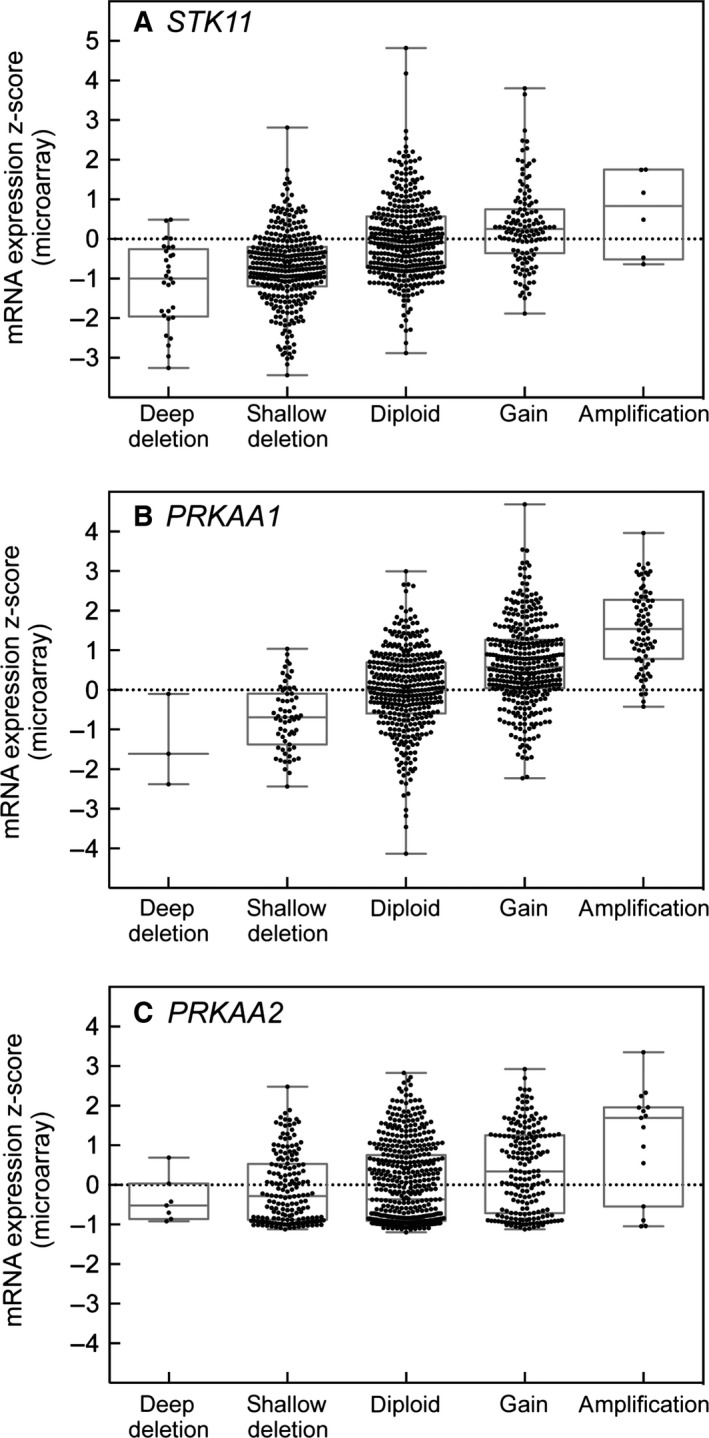
Correlation between deletion or amplification of the (A) *STK11*, (B) *PRKAA1* and (C) *PRKAA2* genes and mRNA expression by micro‐array (*Z*‐scores). ‘Deep’ and ‘shallow’ deletions most likely indicate homozygous and heterozygous gene loss, whereas ‘gain’ and ‘amplification’ indicate moderate and substantial gene amplification, assigned using analysis of single nucleotide polymorphism arrays. Data were from the *Cancer Cell Line Encyclopedia* 68 and were visualized using box and whisper plots; the boxes show the median and the 25th and 75th percentiles, whereas the whispers show the top and bottom values. Note that, with *STK11*, there is a high proportion of ‘shallow’ and a significant proportion of ‘deep’ deletions, which shows some correlation with mRNA expression. By contrast, with *PRKAA1*, there is a high proportion of ‘gains’ and a significant proportion of ‘amplifications’, which also show a clear correlation with mRNA expression. With *PRKAA2*, there is no significant bias towards amplification or deletion or to a change in expression at the mRNA level.

One of the most striking differences between the α1 and α2 isoforms, as recently noted by Monteverde *et al*. 48, emerges from analysis of mutations in the cancer genome databases using cBioPortal 66, 67. As befitting a tumour suppressor, the *STK11* gene encoding LKB1 is often mutated or deleted in cancers (Fig. 3A; note the preponderance of green and blue bars), particularly in lung adenocarcinomas where mutations occur in 15–20% of cases. A majority (> 60%) of the mutations that occur in *STK11* are nonsense or splicing mutations, or insertions or deletions (Fig. 4A, red symbols), all of which are likely to produce nonfunctional proteins. If AMPK did exert the tumour suppressor functions of LKB1, it might have been expected that the AMPK genes would also be either mutated or deleted in different cancers. However, cBioPortal reveals that, in the same cancer genome studies where *STK11* is mutated or deleted, the *PRKAA1* gene encoding α1 is often amplified, with the highest frequency (10–15%) also occurring in lung adenocarcinomas (Fig. 3B; note the preponderance of red bars). By contrast with *STK11*, mutations in *PRKAA1* are infrequent (approximately 0.1% of all cancer cases) and around 80% of those that do occur are missense mutations that may not affect function (Fig. 4B, green symbols). In the current CCLE database 68, which covers almost 900 individual cancer cell lines, *PRKAA1* exhibits a major amplification in 8% and a more moderate gain of gene copy number in 41%, and there is a correlation between gene copy number and mRNA expression (Fig. 5B), suggesting that gene amplification does lead to increased expression. By contrast, the frequency of alterations in the *PRKAA2* gene (encoding α2) in cancer is lower overall (Fig. 3C; note the different scales), and there is no obvious bias towards gene amplification or increased mRNA expression (Fig. 5C). The frequency of mutations is also much higher for *PRKAA2* than *PRKAA1*, although the proportions of missense mutations are quite similar (Figs 3C and 4C). Thus, amplification of the *PRKAA1* gene appears to have been selected for in different cancers, suggesting that it is an oncogene. By contrast, *PRKAA2* is subject to more frequent mutations and, although many might be passenger mutations caused by genomic instability of cancer cells, some may cause a loss of function consistent with the idea that α2 is a tumour suppressor.

There is some independent evidence supporting these divergent roles of α1 and α2 in cancer. As discussed above, a loss of both α1 and α2 in H‐Ras‐transformed MEFs caused a complete failure of their growth *in vivo* in immunodeficient mice 46. Intriguingly, however, although a loss of *PRKAA1* caused failure of MEFs to grow *in vivo* just like the double knockouts, a loss of *PRKAA2* alone caused the tumours to grow more rapidly 69. Taken together with the other findings discussed here, this suggests that, although AMPK‐α2 might indeed represent a tumour suppressor (similar to its upstream kinase, LKB1), AMPK‐α1 may represent instead an oncogene that is frequently amplified in cancers, perhaps because it protects tumour cells against stresses caused by lack of oxygen or nutrients. Our rather limited current insight into the differential roles of the two α isoforms makes it difficult to explain these divergent roles in cancer, and enhancing our understanding in this area represents an important challenge for the future.

### Differences between β1 and β2 (PRKAB1 and PRKAB2)

Although the β1 and β2 subunit isoforms both contain a central CBM, the CBM in β2 (an isoform highly expressed in skeletal muscle, a tissue with high glycogen levels) appears to have a higher affinity for glycogen and glucose oligosaccharides 70. The surfaces of the β‐CBMs opposite to the glycogen‐binding site also contribute to the ADaM site discussed earlier. All of the known allosteric activators that bind this site have a much higher affinity for complexes containing β1 rather than β2 8, 34, 71, although the significance of this difference may remain unclear unless a physiological ligand that binds the ADaM site can be identified.

Similar to the α isoforms, an intriguing difference between β1 and β2 concerns the nature of genetic changes in the respective genes (*PRKAB1* and *PRKAB2*) in the cancer genome databases. Analysis using cBioPortal reveals that changes in *PRKAB1* are relatively infrequent (generally < 4%) and are a mixture of amplifications, deletions, nonsense and missense mutations. By contrast, changes in the *PRKAB2* gene are more frequent (> 10% in many types of cancer) and are almost invariably amplifications 48. Similar to the related findings with the α subunits, it is currently difficult to explain why β2 is selectively amplified, based on our limited knowledge of the functional differences between β1 and β2. In many cancer genome studies, including the *Cancer Cell Line Encyclopedia* 68, the *PRKAA1* and *PRKAB2* genes (encoding α1 and β2) tend to be amplified together (*P* < 0.01), suggesting that there has been selection for amplification of both genes. This may be because the over‐expressed α1 subunit is unstable unless there is an increased level of a β subunit with which it can interact.

### Differences between γ1, γ2 and γ3 (PRKAG1, PRKAG2 and PRKAG3)

The regions of the γ subunits whose function is most well understood are the four tandem CBS repeats at their C‐termini, which, as discussed above, form the binding sites for the regulatory nucleotides AMP, ADP and ATP. In a recent study 18, complexes containing γ1, γ2 and γ3 were expressed in mammalian cells and shown to display potentially important differences in their regulation by adenine nucleotides. First, although γ1 and γ2 complexes were allosterically activated by up to 10‐fold by AMP, γ3 complexes were barely activated at all (< 1.5‐fold). Despite this, all three complexes were activated in intact cells by agents that increased cellular AMP/ADP as a result of increased Thr172 phosphorylation. This suggested that AMP or ADP were either promoting phosphorylation or inhibiting dephosphorylation, and these possibilities were examined in cell‐free assays. AMP caused a four‐ to five‐fold stimulation of activation and Thr172 phosphorylation of γ1 complexes by LKB1, with much smaller effects on γ2 and γ3 complexes. The effect on γ1 complexes was also mimicked by ADP, although only at much higher concentrations. Binding of either AMP or ADP also protected Thr172 against dephosphorylation in cell‐free assays; with γ1 and γ3 complexes, AMP was almost 10‐fold more potent than ADP, whereas, with γ2 complexes, the potencies of AMP and ADP were similar. Thus, the γ1, γ2 and γ3 complexes display interesting differences in the three mechanisms by which adenine nucleotides regulate AMPK 18.

The most striking differences in sequence between the three isoforms occur in their N‐terminal regions. Both γ2 and γ3 contain N‐terminal extensions of up to 240 and 150 residues, respectively, which are unrelated to each other and absent in γ1 (Fig. 2B). The γ2 and γ3 isoforms also occur as various shorter versions: γ3 as one short form with an N‐terminal extension of only 130 residues, and γ2 as the full‐length form (γ2‐a), and three shorter forms that either lack the N‐terminal extension (γ2‐b) or have shorter extensions of approximately 115 (γ2‐c) and 190 (γ2‐3B) residues 72, 73. Most of these appear to be generated via the use of alternate transcriptional start sites, although γ2‐3B, the major form expressed in human heart, is a splice variant containing 32 unique amino acids at the N‐terminus. Although the functions of these variable N‐terminal extensions in γ2 and γ3 remain unclear, it is tempting to speculate that they cause localization of these isoforms at specific subcellular locations. Indeed, the different γ subunit isoforms do appear to localize differently in cardiac and skeletal muscle cells 74. Their precise locations and the proteins that target them to those locations remain largely unknown, although γ1 (despite having only a very short N‐terminal extension) emerged as an interacting protein in a two‐hybrid screen using the cytoskeletal protein plectin as bait, and complexes containing α1 and γ1 localized to the Z disk in wild‐type but not in plectin‐deficient skeletal muscle 75. In human endothelial cells grown in culture, α2, β2 and γ2 appeared to co‐localize at the mid‐body during cytokinesis and were also present in the nucleus, whereas α1, β1 and γ1 were largely cytoplasmic, with little or no nuclear staining 76. The validity of these findings is critically dependent on the specificity of the antibodies used, although they do suggest that different isoform combinations may be present at distinct subcellular locations.

There are also indications that AMPK complexes containing different γ subunit isoforms are regulated differently and have different downstream targets in skeletal muscle. Thus, the AMPK activator AICAR, which is converted to an AMP analogue inside cells, activates glucose uptake in isolated muscles from wild‐type but not AMPK‐γ3^−/−^ knockout mice, suggesting a special role for γ3 complexes in promoting glucose uptake. Consistent with this, the alternative activator PT‐1, which, in contrast to previous studies 77, activates AMPK by inhibiting the mitochondrial respiratory chain and thus increasing cellular AMP, was reported to activate γ1 complexes but failed to activate either γ3 complexes or glucose uptake in isolated muscle, despite the fact that it did activate γ3 complexes expressed in HEK293 cells 78. These results suggest that γ3 complexes are present at a unique location in muscle that is not reproduced in HEK293 cells, where their activity (unlike that of γ1 complexes) is unaffected by increases in AMP resulting from inhibition of mitochondrial respiration. Intriguingly, α2β2γ3 appears to be the only AMPK complex activated by contraction in human skeletal muscle 9. Taking these findings together, the α2β2γ3 complex in muscle appears to be able to sense changes in the AMP : ATP ratio caused by increased ATP turnover by the myosin ATPase but not those resulting from inhibition of mitochondrial ATP synthesis. In muscle, PT‐1 also caused increased phosphorylation of ULK1, an AMPK target involved in regulation of autophagy and mitophagy, but not targets involved in the regulation of glucose uptake (TBC1D1) or fatty acid oxidation (acetyl‐CoA carboxylase, ACACB), both of which were phosphorylated in response to AICAR 78. These results imply that ULK1 is primarily phosphorylated by a γ1 complex that can be activated by mitochondrial dysfunction, whereas TBC1D1 and ACACB are primarily phosphorylated by the α2β2γ3 complex, which is activated instead by muscle contraction. Therefore, different AMPK complexes at distinct locations in skeletal muscle not only respond to different inputs, but also have different outputs.

Finally, from analysis using cBioPortal, it is less obvious (compared to the analysis of α and β subunits described above) that there is any selection for specific alterations of any of the three genes encoding γ subunits in different cancers.

## Conclusions and perspectives

Although much remains to be learned, the results discussed in this review suggest that the numerous heterotrimeric combinations of AMPK subunit isoforms (up to 12, or more when counting variants derived from single genes) may have different subcellular locations, different inputs and outputs, and different functions. A particularly intriguing feature is that genes encoding certain AMPK‐α and ‐β isoforms (i.e. α1 and β2) are frequently amplified together in different cancers, whereas others (i.e. α2 and β1) are more commonly mutated instead. These findings make little sense unless these isoforms have different inputs and/or outputs. Interestingly, except for LKB1 and PTEN all other proteins mentioned in this review, including CAMKK2, GYS1, ULK1, TBC1D1 and ACACB, are also 2R‐ohnologues 79. This raises the question as to whether AMPK variants display selectivity for different sisters in each 2R‐family of regulators, interactors or substrates. Deciphering how different AMPK isoforms are positioned within their sub‐network contexts may be key to understanding their differential roles in cancers 80.

## Author contributions

FAR performed some of the recent experiments covered in the review, and made suggestions for revision of the draft version. CM wrote the first draft of the section on 2R ohnologues, drew Fig. 1, and made many suggestions for improvement. DGH conceived the original idea, wrote most of the first draft and created Figs 2–5.
